# Rac1 modification by an electrophilic 15-deoxy Δ^12,14^-prostaglandin J_2_ analog

**DOI:** 10.1016/j.redox.2015.01.016

**Published:** 2015-02-03

**Authors:** S.B. Wall, J.-Y. Oh, L. Mitchell, A.H. Laube, S.L. Campbell, M.B. Renfrow, A. Landar

**Affiliations:** a Center for Free Radical Biology and Department of Pathology, University of Alabama at Birmingham, AL, USA; b Department of Biochemistry and Molecular Genetics, University of Alabama at Birmingham, AL, USA; c Department of Biochemistry and Biophysics, University of North Carolina, Chapel Hill, NC, USA

**Keywords:** Cyclopentenone, Electrophile responsive proteome, Oxidative post-translational modification, Redox signaling, Rho GTPase, Thiol, 15d-PGJ2, 15-deoxy-Δ^12,14^-prostaglandin J_2_, BAEC, bovine aortic endothelial cells, DMEM, Dulbecco's Modified Eagle's Medium, EC, endothelial cells, EDTA, ethylenediamine tetraacetic acid, FBS, fetal bovine serum, GST-A, glutathione agarose, GST, glutathione-S-transferase, PBS, phosphate-buffered saline, PIC, protease inhibitor cocktail, PBD, protein binding domain, SDS, sodium dodecyl sulfate, Ras-Related C3 Botulinum Toxin Substrate1 Aliases, cell migration-inducing gene 5 protein, TC-25, Ras-like protein TC25, Ras-related C3 botulinum toxin substrate 1, p21-Rac1, TC25

## Abstract

Vascular endothelial cells (ECs) are important for maintaining vascular homeostasis. Dysfunction of ECs contributes to cardiovascular diseases, including atherosclerosis, and can impair the healing process during vascular injury. An important mediator of EC response to stress is the GTPase Rac1. Rac1 responds to extracellular signals and is involved in cytoskeletal rearrangement, reactive oxygen species generation and cell cycle progression. Rac1 interacts with effector proteins to elicit EC spreading and formation of cell-to-cell junctions. Rac1 activity has recently been shown to be modulated by glutathiolation or S-nitrosation via an active site cysteine residue. However, it is not known whether other redox signaling compounds can modulate Rac1 activity. An important redox signaling mediator is the electrophilic lipid, 15-deoxy-Δ^12,14^-prostaglandin J_2_ (15d-PGJ_2_). This compound is a downstream product of cyclooxygenase and forms covalent adducts with specific cysteine residues, and induces cellular signaling in a pleiotropic manner. In this study, we demonstrate that a biotin-tagged analog of 15d-PGJ_2_ (bt-15d-PGJ_2_) forms an adduct with Rac1 in vitro at the C157 residue, and an additional adduct was detected on the tryptic peptide associated with C178. Rac1 modification in addition to modulation of Rac1 activity by bt-15d-PGJ_2_ was observed in cultured ECs. In addition, decreased EC migration and cell spreading were observed in response to the electrophile. These results demonstrate for the first time that Rac1 is a target for 15d-PGJ_2_ in ECs, and suggest that Rac1 modification by electrophiles such as 15d-PGJ_2_ may alter redox signaling and EC function.

## Highlights

• Recombinant Rac1 is modified by bt-15d-PGJ_2_ at C157 in vitro*.*
• Rac1 is modified by bt-15d-PGJ_2_ in bovine aortic endothelial cells.
• Rac1 activity is transiently stimulated by bt-15d-PGJ_2_.
• 15d-PGJ_2_ inhibits endothelial cell migration and spreading.

## Graphical abstract

**Figure:**
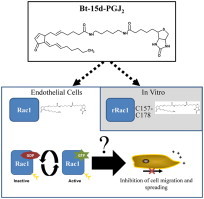

## Introduction

Ras-Related C3 Botulinum Toxin Substrate 1 (Rac1) is a small GTP hydrolyzing (GTPase) protein in the Ras superfamily. Rac1 is ubiquitously expressed in many cell types, and regulation is both dynamic and contextual. Previous studies have described Rac1 dysregulation as a contributing factor in an array of different pathologies including cardiovascular disease and cancer [1–4]. Dysregulation of Rac1 in endothelial cells (ECs) may be important in disease initiation and progression during cardiovascular disease. Many studies have shown that Rac1 functions downstream of many cell surface receptors and is a major pathway by which ECs migrate and align in the direction of flow [5]. In addition, the modulation of EC migration is of interest in vascular restenosis, where normal EC migration and function are necessary for vascular repair after balloon angioplasty and stenting [6]. However, the mechanisms by which Rac1 can be dysregulated by vascular oxidative stress, and therefore contribute to vascular injury, are not clear.

Rac1 acts downstream of G-protein coupled receptors (such as those for sphinogosine-1-phosphate and stromal cell-derived factor-1) and tyrosine kinase receptors (such as those for vascular endothelial growth factor and basic fibroblastic growth factor) and plays a major role in endothelial cell function [7–10]. Similar to other Ras GTPase proteins, Rac1 cycles between an active (GTP-bound) form and an inactive (GDP-bound) form, acting as a molecular switch dependent on the protein’s GTP/GDP bound state [11]. In the GDP bound form, Rac1 is inactive and sequestered in the cytoplasm. Upon binding to GTP, Rac1 undergoes structural changes which allow for the interaction with cell type- specific effector proteins to elicit cellular responses. Rac1 nucleotide binding is mediated by three families of regulatory proteins including guanine exchange factors, guanine activating proteins, and guanine nucleotide dissociation inhibitors [12]. In addition to nucleotide binding, intracellular localization of Rac1 can impact the interaction of Rac1 with its effector proteins near the plasma membrane [13]. Plasma membrane localization is mediated via post-translational modifications of Rac1 including lipidation of cysteine residues. Like other members of the Rho family, Rac1 is lipidated at C189 by the 20-carbon geranylgeranyl group [14,15]. Additionally, the 16-carbon palmitoylation of Rac1 was recently described at C178; this lipidation dynamically regulates the localization of the protein to detergent resistant domains within the plasma membrane [16].

Since the discovery of Rac1, there has been growing interest in the ability of Rac1 to regulate and respond to the cellular reductive and oxidative (redox) environment. Such interest has stemmed from Rac1's direct interactions with enzymes involved in reactive species production and regulation, such as NADPH oxidase, nitric oxide synthase (NOS), and superoxide dismutase 1 (SOD1) [17]. Interestingly, a redox sensitive C18 in the GTP-binding pocket of Rac1 has been described to regulate its activity via both one electron or two electron oxidation mechanisms, via either a thiyl radical-dependent or as most recently described via S-glutathionylation-dependent mechanism [18,19].

An important redox signaling mediator produced during inflammation is the cyclopentenone prostanoid, 15-deoxy-Δ^12,14^-prostaglandin J_2_ (15d-PGJ_2_). This lipid is electrophilic and forms covalent adducts with specific cysteine residues, thereby mediating cellular signaling in a pleiotropic manner [20,21]. 15d-PGJ_2_ has been described to accumulate in human atherosclerotic plaques and promote anti-inflammatory pathways. A well-described target of 15d-PGJ_2_ is the Kelch-like ECH-associated protein 1 (Keap1) which regulates the cytoprotective transcription factor nuclear factor-erythroid 2-related factor 2 (Nrf2) [22,23]. Interestingly, 15d-PGJ_2_ has been observed to have biphasic effects in many cell types including ECs, and up-regulates reactive oxygen species [24]. In particular, 15d-PGJ_2_ has been shown to inhibit migration in endothelial cells [25]. Since Rac1 can be redox-regulated and contributes to migration, we sought to determine whether Rac1 is a cellular target of 15d-PGJ_2_. In particular, this work explores the site specific modification of Rac1 by 15d-PGJ_2_ in vitro and suggests a correlation between Rac1 modification and the inhibition of migration and spreading in endothelial cells. Furthermore, this study highlights some important concepts regarding redox signaling by electrophiles and the role of the target proteins involved in electrophile dependent modulation of biological responses.

## Materials

Primary bovine aortic endothelial cells (BAEC) were collected as previously described [26], or purchased (Lonza, Walkersville, MD). All chemicals were of analytical grade and purchased from Sigma-Aldrich (St. Louis, MO) unless otherwise noted.

## Methods

### Recombinant Rac1 reactions with bt-15d-PGJ_2_

Recombinant Rac1 (rRac1) protein was prepared as previously described [27]. Biotin tagged 15d-PGJ_2_ (bt-15d-PGJ_2_) was synthesized and purified as previously described [28]. rRac1 consisted of residues 1–192 (non-lipidated or cleaved). The protein was stored in 50% glycerol at −20 °C. rRac1 thiols were reduced with 1 mM DTT for 30 min on ice. DTT was removed by dialysis against a Chelex 100 resin (Bio-Rad)-treated buffer solution containing 50 mM Tris–HCl pH 7.5, 150 mM sodium chloride, 50 µM GDP, and 50 mM MgCl_2_ (UltraPure) used for subsequent reactions and assays. The protein concentration of the dialyzed rRac1 was measured by BCA protein assay. Equimolar amounts of rRac1 were reacted with increasing molar ratios of bt-15d-PGJ_2_ (0:1 to 5:1, lipid:protein) for 1 h at room temperature. This was 30 fmols (fmol) of Rac1 with 0.15, 30, or 150 fmol of bt-15d-PGJ_2_ in a total volume of 100 µl. Unreacted bt-15d-PGJ_2_ was quenched using β-mercaptoethanol (β-ME) at a final concentration of 10 µM. Biotinylated lipid adducts on rRac1 were detected either by western blot or mass spectrometry as described below.

### Mass spectrometry of rRac1 adducts

Approximately 1 µg of rRac1 treated with increasing amounts of bt-15d-PGJ_2_ was denatured, reduced, then digested using sequencing grade trypsin (Promega, Madison, WI). Digested peptides were loaded onto a self-prepared 11-cm,100-µm-diameter pulled tip packed with Jupiter 5-µm C18 reversed-phase beads (Phenomenex, Torrance, CA). Samples were analytically separated via nanoLC by use of an Eksigent MicroAS autosampler and 2D LC nanopump (Eksigent, Dublin, CA) via two different methods. For each method, tryptic peptides were separated by liquid chromatography using a gradient of acetonitrile containing 0.2% formic acid and eluted tryptic peptides were electrosprayed at 2 kV into a dual linear quadrupole ion trap Orbitrap Velos (Orbitrap) mass spectrometer (Thermo Fisher Scientific, San Jose, CA). In the first method samples were analyzed making use of collision-induced dissociation (CID), and in the other method, samples were analyzed using high-energy collision dissociation (HCD) for the MS/MS scans. Briefly, the mass spectrometer was set to switch between an Orbitrap full scan (*m*/*z* 300–1800) followed by successive MS/MS scans of the 10 or 15 most abundant precursor ions (parent ions). The dynamic exclusion setting was set to exclude ions for 2 min after a repeat count of three within a 45 s duration. Thermo Xcalibur RAW files were converted to mzXML files using the converter ReAdW program, then to. MGF files with the trans-proteomic pipeline tools software suite. For identification of rRac1 the search engines TurboSEQUEST (Thermo, Fisher Scientific) and MASCOT 2.2 (Matrix Biosciences). SEQUEST and MASCOT searches used the latest available UniRef100 database. Parent ion mass accuracy window was set to 10.0 ppm. For rRac1 modification by bt-15d-PGJ_2_ the search included the mass addition of 626.387 Da on a cysteine residue. SEQUEST searches were processed and visualized using Scaffold (Proteome software Inc., Portland, OR) with the addition of searching with X!Tandem on the Scaffold software. Manual validation of parent ions was aided by the online program Protein prospector (MS-product feature) to produce lists of daughter ions for select peptides and modified peptides. Cysteine containing peptides were searched for as non-modified peptides, carboxyamidomethyl cysteine (CAM; iodoacetamide treatment results in the addition of monoisotopic mass 57.021 to Cys), and bt-15d-PGJ_2_ (addition of 626.387 to Cys).

### Cell culture

BAEC were cultured in Dulbecco's modified Eagle's medium (DMEM, Cellgro, Herndon, VA) containing 10% fetal bovine serum (FBS, Atlanta Biologicals, Atlanta, GA) and supplemented with 5.6 mM d-glucose, 4 mM glutamine and 100 μ/ml penicillin and 100 µg/ml streptomycin (Gibco, Grand Island, NY). Cells were used between passage 5 and 9 and were cultured in 6-well plates to confluency. For treatments, media was replaced with low-serum DMEM (0.5% FBS) for 16 h prior to addition of either 15d-PGJ_2_ or bt-15d-PGJ_2,_ as described previously [29]. Treatment with lipid is expressed as a concentration but may be converted to amount lipid per cell, since in all experiments 2 ml of media is used in 6-well plates with confluent BAEC; we estimate that, the treatment of 10 µM bt-15d-PGJ_2_ is equivalent to approximately 20 fmol/cell in our conditions. Unless noted otherwise, cells were washed once prior to treatment with ice cold phosphate buffered saline (PBS) and lysed with RIPA lysis buffer [50 mM Tris–HCl, pH 7.4, 0.5% w/v sodium deoxycholate, 0.1% w/v SDS, 150 mM NaCl, 1 mM ethylenediamine tetraacetic acid (EDTA), and 1% v/v NP-40), and protease inhibitor cocktail (PIC, Roche Diagnostics, Indianapolis, IN). Lysates were cleared by centrifugation at 16,862*g* unless noted otherwise. Protein concentrations were measured by DC Lowry protein assay (BioRad, Hercules, CA) adapted for microplates. Cell viability was measured by lactate dehydrogenase release assay as previously described [30].

### Biotin affinity precipitation

Affinity precipitation of biotin was performed using Neutravidin resin. Briefly, BAEC were treated at various times and concentrations of bt-15d-PGJ_2_ as described in the results. After treatment, BAEC were lysed and cleared by centrifugation. Approximately, 25 µl settled resin (50 µl of 50% slurry; Thermo Scientific) was used for each experiment and was loaded into Micro Bio-Spin columns (Bio-Rad, Hercules, CA). The resin was equilibrated with RIPA buffer prior to loading protein according to the concentration of bt-15d-PGJ_2_ (see Table S2 for resin load information). Volumes were adjusted with RIPA lysis buffer to normalize protein concentration to 1 mg/ml and incubated overnight at 4 °C on a shaker. Resin was washed six times with 200 µl volumes of RIPA lysis buffer. Resin was transferred to a fresh tube in three volumes of 200 µl RIPA and allowed to settle by gravity, and supernatant was removed. Bound proteins were eluted by heating at 80 °C for 10 min in 1.5 fold volume of 2× sample buffer (0.1 M Tris–HCl, 4% SDS, 10% Glycerol, 0.2% Bromophenol blue, pH 6.8, containing 2% β-mercaptoethanol) on settled resin by heating at 80 °C for 10 min. Within 5 min after heating, resin was briefly centrifuged and supernatants were collected to a fresh tube for analysis by SDS-PAGE or stored at −20 °C for later analysis.

### Rac1 activity assay

A plasmid encoding a glutathione-S-transferase fusion protein with Rac1 p21 protein binding domain (GST-PBD) was a generous gift from Dr. Rakesh Patel (University of Alabama at Birmingham), and protein was expressed and purified as previously described [31]. The Rac1 activity assay was performed based as previously described with minor changes [32]. Briefly, BAEC were grown to 80% confluence and media replaced with low-serum DMEM (0.5% FBS) for 16 h prior to treatment with 7.5 µM bt-15d-PGJ_2_. At indicated time points, cells were washed once with PBS and lysed with 100 µl per well of Rac activity lysis buffer [50 mM Tris (pH 7.4), 150 mM NaCl, 0.5% NP-40, 10% glycerol, 1 mM phenylmethylsulfonyl fluoride (PMSF), and PIC] for 5 min. Samples were collected and centrifuged at 10,000*g* for 5 min. Lysate (80 µl) was added to 25 µg of GST-PBD and 20 µl of 50% glutathione agarose (GSH-A) slurry. This mixture was incubated for 30 min at 4 °C on a shaker. GSH-A was washed with 40 µl (4 times volume of the settled resin) lysis buffer three times. After the final wash, active Rac1 was eluted from GSH-A with 10 µl of 2× sample buffer with β-ME. The total 2 µl including resin was loaded onto a 12.5% SDS-PAGE gel and 2 µl of each reserved 20 µl of comparable lysate was loaded as an input control to compare to the eluate. For negative and positive controls, respectively, GDP (1 mM) or non-hydrolyzable GTP (γ-S-GTP; 0.1 mM) was added to the pre-cleared lysates and processed the same as all samples. To calculate percent active Rac1, western blot pixel density of the 21 kDa GST-PBD eluted Rac1 (active Rac1) was expressed as a percentage of total Rac1 in the input (total Rac1 in lysate).

### Cell migration assay

Cell migration was assessed as previously described [33]. Briefly, BAEC were grown to 80% confluence in 6-well plates. Media was replaced with low-serum DMEM (0.5% FBS) for approx. 16 h. A sterile pipet tip was used to form 4 cell-free zones in a hash tag pattern into the confluent monolayer of cells. Media was immediately changed to fresh low-serum DMEM to remove floating cells and debris, and cells were treated with the indicated concentrations of 15d-PGJ_2_ or ethanol vehicle control for 4 h before the cell media was changed to fresh low-serum media; following this, cells were allowed 4 additional hours to continue migrating (8 h total migrating time). The width of the cell-free zone was measured immediately after scratching for an initial measurement, and again after 8 h at 37 °C in order to allow cell migration. The migratory ability of the lipid treated cells into the cell-free zone was expressed as percent change in the width of the scratch compared to the vehicle controls at 8 h (relative motility).

### Cell spreading assay

Cells were seeded at a density of 2×10^5^ cells/well and allowed to reach 70–80% confluence in 10% serum DMEM. Media was changed to low-serum DMEM (0.5% FBS) for 16 h, then cells were treated with bt-15d-PGJ_2_ or EtOH vehicle for 4 h. Cells were detached by 0.05% Trypsin (Gibco, Grand Island, NY) for 3 min, followed by neutralization of trypsin with 1 ml of DMEM (10% FBS). Cells were pelleted and resuspended in DMEM (10% FBS). Cells were replated at a threefold dilution and were evaluated after both 30 min and 2 h. For each condition, 4 images were acquired and analyzed by counting the total number of cells per field (10× magnification), and the number of cells spread/field. Data are expressed as cells spread (%) and values were calculated as (number of cells spread/total number of cells)×100.

### Western blot analysis

For all lysate samples, protein concentrations were measured via Lowry, DC and loaded by equal protein amounts onto 12.5% SDS-polyacrylamide gel electrophoresis (SDS-PAGE) and transferred to nitrocellulose. Transferred protein and amount loaded was confirmed with Ponceau S stain. Membranes were blocked with 5% nonfat milk in TBS-T. Rac1 antibody (Millipore, Billerica, MA) or β-actin antibody (Cell Signaling) was used at a dilution of 1:3000 or 1:5000, respectively, in 0.5% milk TBS-T. Anti-mouse IgG-HRP (Amersham, Amersham, UK) secondary was used at 1:10,000 in TBS-T for Rac1 and Anti-rabbit IgG-HRP (Amersham) secondary antibody was used at 1:10,000 in TBS-T for β-actin. In order to detect biotin, blots were incubated with 10 ml of Streptavidin-HRP (1:10,000 in TBS-T) for 1 h. HRP-conjugated signal was visualized via chemiluminescence using SuperSignal West Dura (Thermo Scientific, San Jose, CA), detected using a FluorChem M camera imaging system (ProteinSimple). Protein bands were quantified by analysis of pixel density using Alpha ViewSA version 3.4.0 software (ProteinSimple, San Jose, CA).

### Statistical analysis

Data are reported as mean±S.E.M. and sample sizes indicated in the legends. Statistical significance was evaluated by either standard two-tailed student *t*-test or via one-way or two-way ANOVA (analysis of variance) among the groups using Graph pad (version 5.0). The minimum level of significance was set at *p*<0.05. The least significant difference (LSD) test was used as a post hoc test for the significant difference among the groups.

## Results

### Determination of Rac1 modification by 15d-PGJ_2_ in vitro

**Fig. 1:**
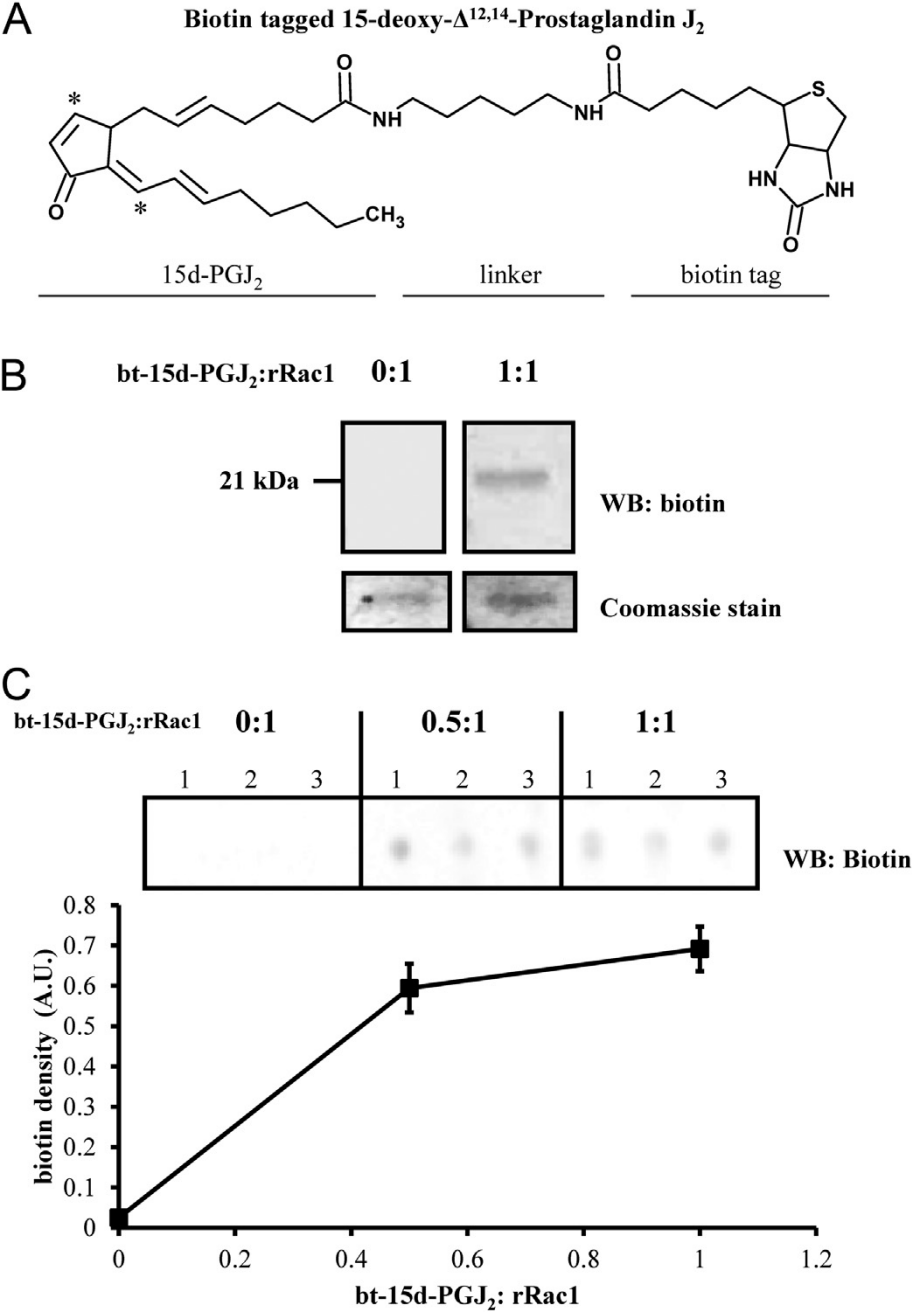
Modification of recombinant Rac1 by biotin-tagged 15-deoxy-Δ^12,14^-Prostaglandin J_2_. (A) The chemical structure of bt-15d-PGJ_2_ showing the electrophilic carbons (indicated by asterisks), a five-carbon linker region flanked by amide bonds, and a biotin tag. (B) Recombinant Rac1 was reacted with either vehicle (0:1) or 1:1 M ratio of bt-15d-PGJ_2_:rRac1 for 1 h at room temperature. Modified rRac1 was detected by Western blot analysis for biotin. Total rRac1 was analyzed by Coomassie stain. (C) Recombinant Rac1 (rRac1) was reacted with increasing molar amounts of bt-15d-PGJ_2_ at the indicated ratios. Reaction mixtures were loaded directly onto nitrocellulose via a dot blot apparatus and biotin signal was detected by Western blot. Biotin density was plotted for each reaction condition (*n*=9 in one independent experiment).

In order to follow modification of proteins by 15d-PGJ_2_, a biotin-tagged analog (bt-15d-PGJ_2_) was used. The chemical structure is shown in Fig. 1A. The tagged analog consists of the parent 15d-PGJ_2_ molecule, a linker region and a biotin tag. Bt-15d-PGJ_2_ forms covalent adducts to cysteine residues via the two electrophilic carbons (indicated by asterisks), and protein adducts have been detected using the biotin tag [29,34–38]. Purified recombinant Rac1 (rRac1) protein was used to assess modification by bt-15d-PGJ_2_ in vitro. When rRac1 was treated with equimolar amounts of bt-15d-PGJ_2_, a biotin signal was detected by western blot at the expected molecular weight of rRac1 (21 kDa), whereas no signal was detected in the untreated rRac1 (Fig. 1B). The treated rRac1 was also applied to nitrocellulose by dot blot in order to determine the protein modification by increasing lipid concentration. Interestingly, rRac1 modification by bt-15d-PGJ_2_ did not increase linearly with concentration, since maximal modification occurred at 0.5:1 (bt-15d-PGJ_2_: rRac1) (Fig. 1C).

### Identification of sites of Rac1 modification in vitro by mass spectrometry

To determine the sites of adduct formation on rRac1, rRac1 was treated with bt-15d-PGJ_2_ (1:1 to 1:5 ratio, mol protein:mol lipid) and then analyzed by a series of high resolution mass spectrometry experiments to identify peptide adducts. After reacting rRac1 with lipid, the tryptic peptides were subjected to liquid chromatography–tandem mass spectrometry (LC–MS/MS; as described in the methods). Fragmentation of peptides was accomplished using collision induced dissociation (CID) or high-energy collision dissociation (HCD) from the same samples in order to maximize protein coverage. The high resolution LC–MS/MS analyses were searched by use of the SEQUEST and MASCOT search algorithms. As expected, the protein was identified as human Rac1 (UniProt entry P63000) using both search engines (Table S1). Table S1 details the peptide fragmentation method, percent coverage, and search scores (for potential bt-15d-PGJ_2_ peptide adducts) for each reaction condition. These results also indicated modification by bt-15d-PGJ_2_ occurs on two cysteine residues of Rac1.

**Fig. 2:**
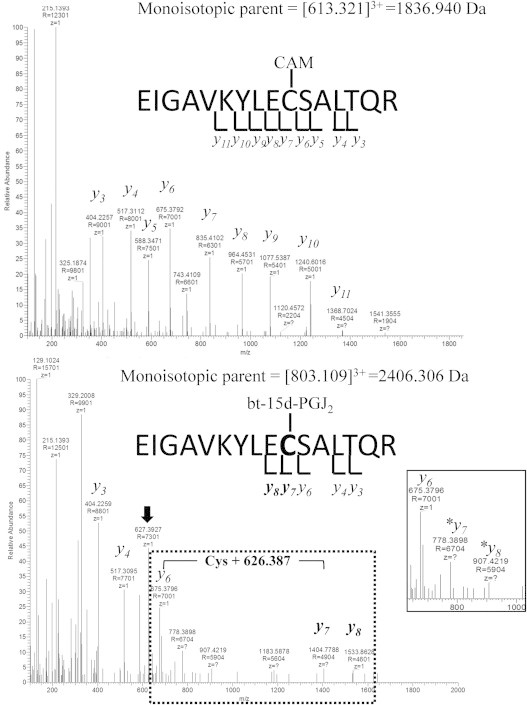
Mass spectrometric identification of C157 as a site of rRac1 modification. rRac1 was reacted with a 1:1 molar ratio of bt-15d-PGJ_2_:rRac1 for 1 h at room temperature. Reaction mixtures were alkylated (carboxyamidomethylated) and subjected to tryptic digestion, separated via HPLC and detected and analyzed via high resolution mass spectrometry with data dependent selection of m/z for tandem mass spectrometry. The rRac1 peptide containing C157, EIGAVKYLECSALTQR, was observed in both an alkylated form (top spectrum) and an adducted bt-15d-PGJ_2_-modified form (bottom spectrum). The top panel shows the alkylated (carboxyamidomethylated; CAM) peptide indicated by a series of *y*-ions. The CAM adduct is indicated on C157. The bottom panel shows bt-15d-PGJ_2_-adducted peptide indicated by a series of *y*-ions, showing the mass gap of Cys+626.387 Da (bt-15d-PGJ_2_) within the dotted box. The inset contains a close-up of the region of the m/z spectrum from 650 to 1000. ^⁎^*y*7 and ^⁎^*y*8 indicate the absence of the Cys+626.387 Da mass gap, and the black arrow indicates the *m*/*z* of 627.393, consistent with [bt-15d-PGJ_2_]^+1^, as described in Results.

In order to further investigate these cysteine adducts, high resolution broad band mass spectra (MS1) features were examined and manual interpretation of the MS/MS spectra was done using theoretical MS/MS peak lists with the Protein Prospector MS Product tool. Fig. 2 shows tandem mass spectra of parent ions from carbamidomethyl ([613.321]^3+^, top spectrum) and bt-15d-PGJ_2_ adducts ([803.109]^3+^, bottom spectrum) on the C157 containing peptide (^148^EIGAVKYLEC^157^SALTQR^163^) obtained from the 1:1 reaction by HCD. Each spectrum shows the relative abundance of the daughter ions for the respective parent ion. The parent ion, [613.321]^3+^ (monoisotopic mass 1836.940 Da), was the exact mass addition of carbamidomethyl to C157, indicating a carbamidomethyl adduct (CAM) formed by reaction of Rac1 with iodoacetamide. Daughter ions were assigned to the peptide via a sequential series of singly charged *y*-ions, *y*3–*y*11 (Fig. 2, top panel). Additionally, another observed parent ion, [803.109]^+3^ (monoisotopic mass 2406.306 Da) was the exact mass of bt-15d-PGJ_2_ to C157 (Fig. 2, bottom panel). Three daughter ions (*y*3, *y*4, and *y*6) with similar mass were found from both spectra. Furthermore, the daughter ions, *y*6, *y7* and *y*8 indicated that the bt-15d-PGJ_2_ adduct occurred on C157 (indicated by the mass gap in the dotted box). Interestingly, the presence of bt-15d-PGJ_2_ is confirmed by daughter ion [627.393]^+1^ which corresponds to protonated bt-15d-PGJ_2_ and is marked by an arrow. Additionally, two *y − ions (*y7 and *y8 in the insert) correspond to the *y*7 and *y*8 peptides without the bt-15d-PGJ_2_ adduct. This implies that the fragmentation energy was sufficient to remove the bt-15d-PGJ_2_ adduct from the peptide.

A potential adduct on the C178 peptide was identified in the MS1 high resolution spectra, but we were unable to verify the addition by MS/MS. Several proteolytic fragment ions were detected with masses consistent with addition of bt-15d-PGJ_2_. Manual interpretation of the MS/MS spectra could only identify the peptide sequence, but the fragmentation was not sufficient to unambiguously assign the bt-15d-PGJ_2_ adduct on the cysteine (data not shown). These results suggest that C178 is likely a target of adduct formation due to the unique mass of the adduct formed, but this site could not conclusively be confirmed. The fact that other sides of adduct formation were not detected does not rule out these sites, but if they were modified it was well below our limit of detection.

### Modification of Rac1 in endothelial cells

Our group and others have previously demonstrated that 15d-PGJ_2_ modifies a discreet electrophile responsive proteome in multiple cell types [29,39,40]. However, whether 15d-PGJ_2_ modifies Rac1 in endothelial cells is not known. Primary bovine aortic endothelial cells (BAECs) were treated for 24 h with increasing concentrations of 15d-PGJ_2_. Cytotoxicity was not observed up to 5 µM 15d-PGJ_2_ (10 fmol/cell), whereas viability decreased to 78% and 24% at 10 and 20 µM 15d-PGJ_2_ (20 and 40 fmol/cell), respectively (Fig. S1). These results are consistent with our previous reports of 15d-PGJ_2_ effects on viability in endothelial cells [30].

**Fig. 3:**
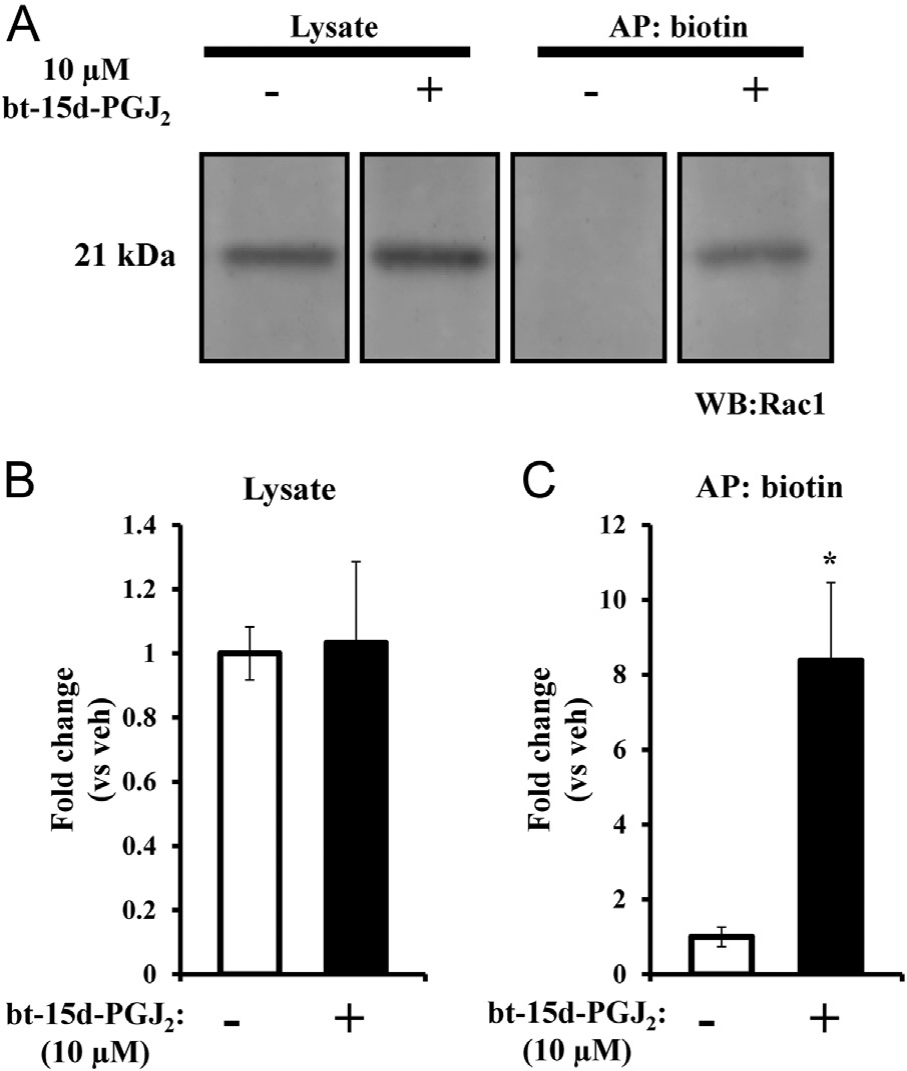
Modification of Rac1 in endothelial cells. BAEC were treated with 10 µM bt-15d-PGJ_2_ for 4 h. Lysates were collected and subjected to biotin affinity precipitation. (A) Representative blot for Rac1 showing vehicle or treated lysate and biotin affinity precipitation eluate (AP:biotin). Panels are taken from the same image and cropped for ease of presentation. Bar graphs show the average fold change compared with vehicle control in lysate (B) or after biotin affinity precipitation (C) (±SEM, *n*=4; ^⁎^=*p*<0.05).

Rac1 modification in endothelial cells was assessed by affinity precipitation using the biotin analog of 15d-PGJ_2_, bt-15d-PGJ_2_. We have previously shown that bt-15d-PGJ_2_ elicits similar cellular effects as 15d-PGJ_2_ at similar concentrations in low serum conditions [28]. Fig. 3 shows that total Rac1 levels did not change in response to the lipid over 4 h (Fig. 3A and B), and Rac1 was only detected by affinity precipitation in the presence of bt-15d-PGJ_2_ (Fig. 3A and C), demonstrating that Rac1 is modified by the lipid. These data represent the first evidence that the GTPase protein, Rac1, is modified by 15d-PGJ_2_ in endothelial cells.

**Fig. 4:**
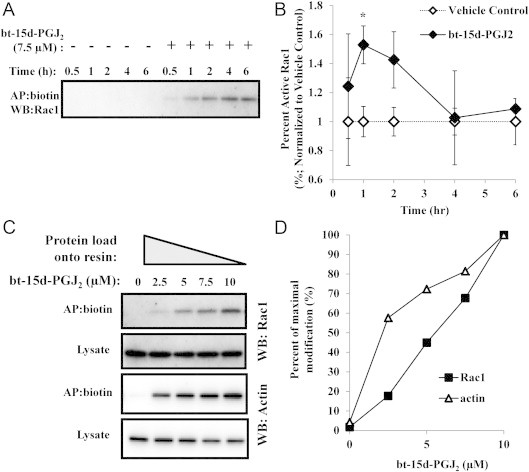
Time- and dose-dependent modification of Rac1 in endothelial cells by bt-15d-PGJ_2_. (A) BAEC were treated with 7.5 µM bt-15d-PGJ_2_ (+) or vehicle control (−) for 0.5, 1, 2, 4, and 6 h, followed by biotin affinity precipitation, and Rac1 was detected by Western blot analysis. (B) Rac1 activity was determined at each time point and normalized to vehicle control (expressed as percent active Rac1 of the total Rac1±SEM; ^⁎^=*p*<0.05; *n*=3; except at 4 and 6 h where only two replicates were done). (C) Biotin affinity precipitation was performed on endothelial lysates after treatment with the indicated concentrations of bt-15d-PGJ_2_. Rac1 and β-actin were detected by Western blot analysis. The protocol was adjusted to compensate for differences in bt-15d-PGJ_2_-adducted proteins by increasing the protein load onto the affinity resin (see Methods for further details). (D) The percent of maximal modification of Rac1 and β-actin (determined at 10 µM bt-15d-PGJ_2_) was plotted for the treatment conditions in the AP:biotin blots from Panel C.

To assess the Rac1-bt-15d-PGJ_2_ adduct formation over time, BAEC were treated with 7.5 µM bt-15d-PGJ_2_ (15 fmol/cell) for 0.5–6 h. Rac1 modification occurred as early as 0.5 h and increased over time until 6 h (Fig. 4A). Over the same time frame and treatment conditions, we sought to determine whether Rac1 activity was altered. Interestingly, Rac1 activity increased significantly at 1 h post-treatment, and at 2 h began to return to control levels (Fig. 4B). This activation initially coincided with formation of Rac1 bt-15d-PGJ_2_-adduct formation; however, at 4 h where maximal adducts were observed, the activity was not different to control levels. Taken together, these results suggest cellular regulation of Rac1 activity by bt-15d-PGJ_2_ occurs at relatively early time points, and is not directly proportional to adduct formation by bt-15d-PGJ_2_.

It is known that bt-15d-PGJ_2_ protein adduct formation increases in a concentration- and time-dependent manner [29]. However, it is not known whether the pattern of protein adducts changes as a function of bt-15d-PGJ_2_ concentration. Therefore, adducts were compared over a range of lipid concentrations (0–10 µM) in biotin affinity precipitated lysates (see Table S2). In this method, the amount of lipid adducts affinity precipitated is similar across a range of concentrations, allowing for a more direct comparison of biotin signal patterns at different amounts of the lipid. No change was observed in the adduct banding pattern by western blot with increasing amounts of bt-15d-PGJ_2_ (Fig. S2). Next, Rac1 protein levels were measured after affinity precipitation from cells treated with bt-15d-PGJ_2_. Fig. 4C shows increased modification of Rac1 with increasing bt-15d-PGJ_2_ concentration, although the treatment did not change the total Rac1 in lysates. Since bt-15d-PGJ_2_ has previously been shown to modify cytoskeletal proteins such as β-actin [38], the degree of Rac1 modification was compared to β-actin in our study. Fig. 4C shows that β-actin was also modified in an increasing manner up to 10 µM lipid. Bt-15d-PGJ_2_-modified Rac1 or β-actin was expressed as a percentage of modification from 10 µM then plotted as a function of bt-15d-PGJ_2_ (Fig. 4D). This plot suggests that Rac1 modification increases in a linear manner up to 1 µM bt-15d-PGJ_2_, whereas a higher percentage of β-actin protein is modified by bt-15d-PGJ_2_ at lower concentrations_._

**Fig. 5:**
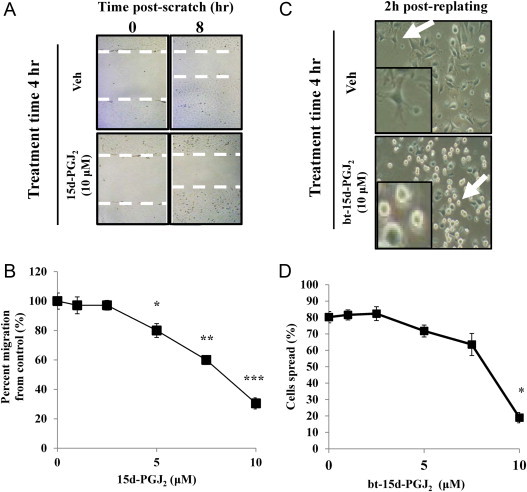
Endothelial cell migration and spreading in response to 15d-PGJ_2_ or bt-15d-PGJ_2_. (A) A confluent monolayer of BAEC was wounded by scratching. Medium was immediately changed and cells were treated with vehicle or 10 µM 15d-PGJ_2_. At 4 h after treatment, medium was changed again to remove lipid, and an additional 4 h was given for cells to migrate (8 h total) before assessing scratch width. Representative images of scratch zone are shown. Cell migration fronts are indicated by white dotted lines. (B) Cell migration into the cell free zone was determined for 1–10 µM of 15d-PGJ_2_, and expressed as a percent of cell migration of vehicle-treated cells (±SEM; *=*p*<0.05; **=*p*<0.005; ***=*p*<0.0005; *n*=4). (C) BAEC were treated with vehicle or bt-15d-PGJ_2_ (1–10 µM) for 4 h. Cells were replated as described in the methods, and imaged after 2 h for assessment of cell spreading. Representative images (20×) of cells for vehicle and 10 µM 15d-PGJ_2_-treated cells are shown. White arrows indicate cells magnified in the inset. Cell spreading was assessed in four frames per condition. (D) Cells spread were quantified for each bt-15-PGJ_2_ concentration (expressed as percent cells spread ± SEM; *=*p*<0.001; *n*=8 from two independent experiments).

Since Rac1 activity was altered in response to bt-15d-PGJ_2_ adduct formation, the effects of the electrophile on endothelial cell functions which are downstream of Rac1 were determined. First, endothelial cell migration was monitored in response to 15d-PGJ_2_ using a scratch assay. Shown in Fig. 5A are representative images of vehicle and 10 µM 15d-PGJ_2_-treated BAEC just after scratching and after 8 h. The migration of endothelial cells was inhibited significantly by 5–10 µM 15d-PGJ_2_ (Fig. 5B). Next, Rac1 is crucial for the formation of cellular protrusions (e.g. lamellipodia) such as those formed by adherent cells just after replating. Therefore, cell spreading was assessed in response to bt-15d-PGJ_2_. Fig. 5C shows representative images 2 h after replating. An example of a spread cell is marked a white arrow and is shown in the inset having a protrusion(s) extending from the cellular body. Cells treated with bt-15d-PGJ_2_ which had not spread were characterized by a round shape with a bright halo. The percent cells spread was plotted as a function of increasing concentrations of bt-15d-PGJ_2_ in Fig. 5D. Significant inhibition of cell spreading was observed at 10 µM bt-15d-PGJ_2_ compared to vehicle control.

## Discussion

Electrophilic lipids are signaling mediators which elicit a coordinated biological response through the direct modification of protein nucleophiles. It is becoming increasingly clear that electrophilic lipids have additional advantages as signaling mediators since they form covalent adducts allowing the effect of adduction to persist and accumulate over time. In particular the electrophilic lipid, 15d-PGJ_2_, is a cyclooxygenase 2 (Cox2)-derived electrophilic lipid which is formed endogenously downstream of PGD_2_, and has been described as an anti-inflammatory mediator in macrophages and other cell types [39,41,42]. 15d-PGJ_2_ has also been found in atherosclerotic lesions colocalized with Cox2 [41]. Biological responses of 15d-PGJ_2_ have been reported to occur due to multiple mechanisms of action and via direct modification of protein thiols [40]. The formation of these protein adducts has been hypothesized to elicit specific biological responses via coordinated adduction to the electrophile responsive proteome [43]. Some previously described members of 15d-PGJ_2_ modified proteome are β-actin, Keap1, and H-Ras. For example, Renedo et al. reported that two electrophilic lipids, PGA_1_ and 15d-PGJ_2_, modified H-Ras at two different cysteine residues [44]. Interestingly, modification of H-Ras by 15d-PGJ_2_ at C184 in the c-terminal hypervariable domain may alter palmitoylation of the protein and may thereby alter downstream H-Ras function via interacting with effector proteins [35]. The fact that H-Ras is modified by bt-15d-PGJ_2_ suggests that other GTPase proteins such as Rac1 may also be targets for the lipid. In fact, Rac1 is poised to be an important mediator of redox signaling, due to its role in a wide variety of cellular functions including reactive oxygen species formation via Nox, cellular migration, and modulation of downstream signaling pathways.

Rac1 contains seven Cys residues which are highly conserved across species and therefore, may have important physiological functions. The C189 residue on Rac1 has been well characterized, and has been demonstrated to be a site of geranylgeranyl addition. This modification occurs in the endoplasmic reticulum immediately following protein translation as a permanent modification, suggesting that this site not available for redox signaling. It was recently shown that Rac1 is palmitoylated at C178 [16]. Unlike geranylgeranyl modification, palmitoylation is a reversible lipidation which allows for the transient association of a protein to detergent resistant membranes and is thereby an important regulator of localization [45,46]. Interestingly, Navarro-Lérida et al. further demonstrated that inhibiting palmitoylation by mutation C178S decreased cellular migration. Another cysteine residue of Rac1, C18, is located within the guanine nucleotide binding pocket. This residue has been shown to be modified by the nonradical oxidant glutathione, and guanine nucleotide dissociation has been shown to be increased both S-glutathionylation and by radical oxidants such as •NO_2_ and superoxide [18,19,47]. C18 was not found to be modified by 15d-PGJ_2_ in our studies. Modification at C18 by 15d-PGJ_2_ cannot fully be excluded, since tryptic peptides containing C18 were very large (>20 residues in length), and therefore, the addition of a lipid may have prevented the modified peptide from being detected. Although this residue has a relatively high solvent accessibility compared with other cysteines [47], 15d-PGJ_2_ is a relatively large hydrophobic molecule and may be sterically hindered from interaction with residues within the binding pocket, or may preferentially interact with more hydrophobic regions of the protein.

In this study, we show for the first time that C157 and C178 of Rac1 are modified by 15d-PGJ_2_ in vitro. C157 is located in a loop between the sixth β-strand and the α5 helix (referred to as the G-5 region) [11]. It has recently been shown that mutation of C157, a residue unique to Rho proteins, impairs the interaction of the Rho mutant protein with its guanine nucleotide exchange factor [48]. However the precise role of C157 in Rac1 function in endothelial cells is still not clear. The other modification observed in our study was at C178. This site has been shown to be palmitoylated. Interestingly, previous studies showed that mutation of this residue inhibited Rac1 localization, reduced spreading and delayed cellular migration [16]. Under our conditions (7.5 µM bt-15d-PGJ_2_) we found that Rac1 activity was increased at 1 h, but returns to baseline at 2 h (Fig. 4B). The fact that overall protein adducts increase over this time scale (Fig. 4A), suggests that increases in Rac1 modification and Rac1 activity are disproportionate. It is intriguing to speculate based on our recombinant studies, that there may be two distinct regulatory sites acting as allosteric mediators of Rac1 activity in response to bt-15d-PGJ_2_ in endothelial cells. While intriguing, the studies herein only determine modification sites in the recombinant protein which may not reflect modifications within the cell. Therefore, experiments to test site specific modification in cells are necessary to correlate Rac1 modification and activity with changes in cell migration or spreading. Ideally, the role of C157 and C178 modification would be investigated using mutational studies. However, Rac1 Cys-mutations produce non-functional protein as the previous studies found with the C157R and C178S mutants [16,48]. Therefore, inhibitory effects of modifications at these sites are not likely to be interpretable.

The overall effects of 15d-PGJ_2_ on cells is dependent on several factors, including amount of lipid, time of exposure, and cell type and presence of effector proteins. It has been reported that subnanomolar concentrations of 15d-PGJ_2_ can stimulate cellular migration of eosinophils in response to a chemoattractant [49]. Other studies report inhibition of cellular migration in multiple cell types in response to micromolar levels of 15d-PGJ_2_ [33,50]. This biphasic phenomenon of 15d-PGJ_2_ may be explained by the accumulation of 15d-PGJ_2_ protein adducts on specific target residues over time, as described by Oh et al. [29]. In addition, it is known that adduct formation occurs on a number of target proteins simultaneously, and therefore, the observed inhibition of migration and spreading in response to 15d-PGJ_2_ may not be solely due to Rac1 modification. In fact, as noted in our studies, other cytoskeletal proteins such as β-actin are sensitive targets for 15d-PGJ_2_ modification [33,36,51]. Currently, methods of studying electrophile-proteomes are limited. Based on what we know, cytoskeletal proteins including those in the Ras superfamily may be important members of this proteome.

In conclusion, we have shown that rRac1 residues C157 and C178 are modified by bt-15d-PGJ_2_. Adduct formation with other cysteine residues, particularly C18 which is a likely target for redox modification, cannot be ruled out by these results. Altogether, these results suggest that modification of Rac1 by electrophilic lipids may be an additional axis of Rac1 regulation not previously described, and future studies are warranted.

## Appendix A Supplementary materials

Fig. S1 Cell viability in response to 15d-PGJ_2_. BAEC were treated with indicated concentrations of 15d-PGJ_2_ for 24 h and cell viability was measured by lactate dehydrogenase (LDH) release assay (percent viable cells ± SEM; *n*=3; *=*p*<0.001).

Fig. S2 Assessment of the total bt-15d-PGJ_2_ adducted proteome using an adjusted affinity precipitation procedure. Affinity precipitation was performed to assess total protein modification by bt-15d-PGJ_2_ at various concentrations of the lipid. The protocol was adjusted to compensate for differences in bt-15d-PGJ_2_-adducted proteins by increasing the protein load onto the affinity resin (see methods for further details). Western blot for total modified protein at each concentration of the lipid (top panel). Asterisks indicate endogenous biotin containing protein. Ponceau stain of the biotin blot shown above (bottom panel).

Table S1 Mass spectrometry experiment summary. Results from mass spectrometry experiment performed on two different dates are shown in the table. Differences in methods are indicated. Collision induced dissociation (CID) was performed on each date with the same liquid chromatography gradient profile. High collision dissociation (HCD) was performed with a different liquid chromatography gradient profile.

Table S2 Concentration-dependent bt-15d-PGJ_2_ affinity precipitation table. For each condition of bt-15d-PGJ_2_, the protein load onto 25 µl of settled resin was adjusted according to the fold difference from the highest concentration of lipid used in the study.
