# Supplementary figures and images for: Rac1 modification by an electrophilic 15-deoxy Δ12,14-prostaglandin J2 analog
Source: Redox Biol. 2015 Feb 3;4:346–54. doi: 10.1016/j.redox.2015.01.016 (PMC4326178; doi:10.1016/j.redox.2015.01.016)

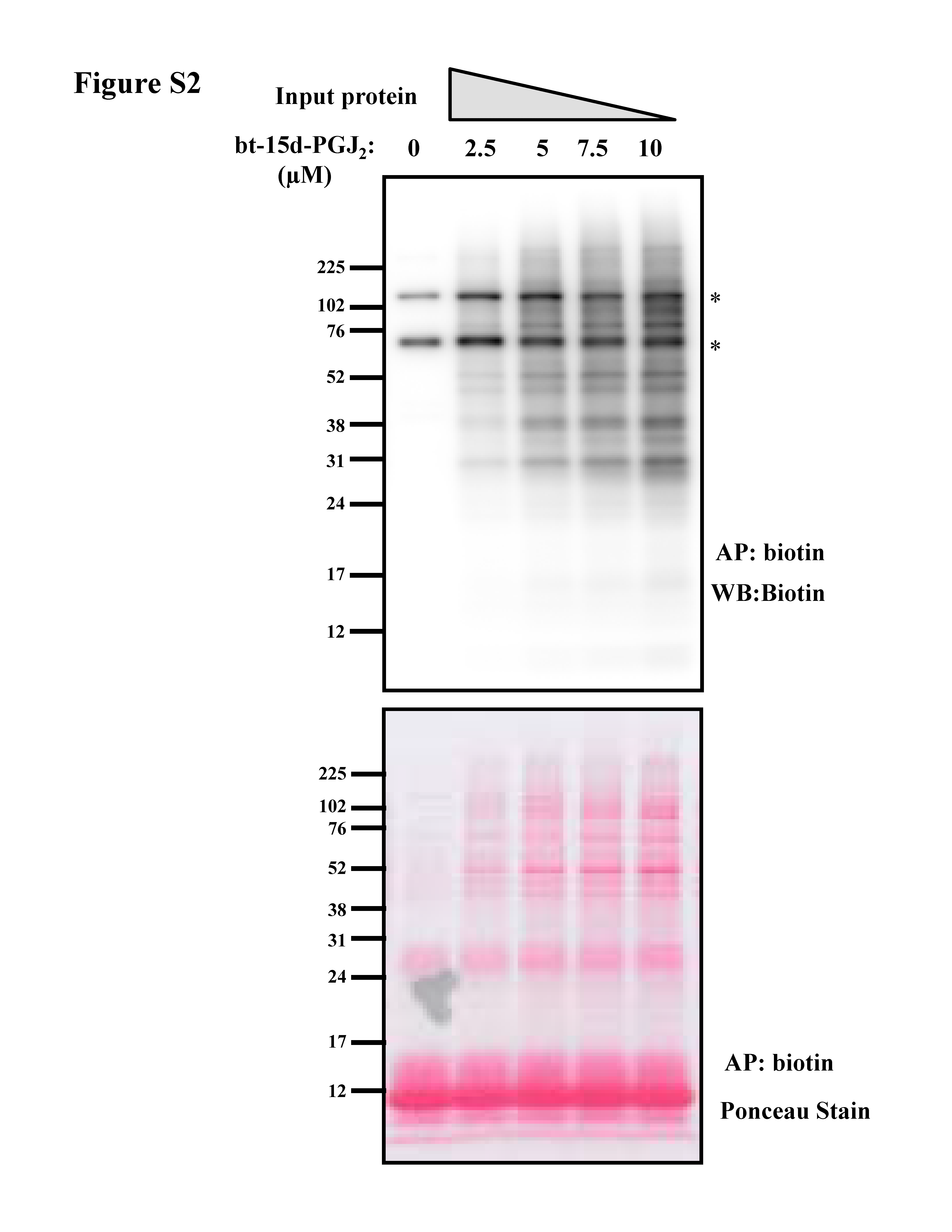

Supplement: Fig. S2 — Assessment of the total bt-15d-PGJ2 adducted proteome using an adjusted affinity precipitation procedure. Affinity precipitation was performed to assess total protein modification by bt-15d-PGJ2 at various concentrations of the lipid. The protocol was adjusted to compensate for differences in bt-15d-PGJ2-adducted proteins by increasing the protein load onto the affinity resin (see methods for further details). Western blot for total modified protein at each concentration of the lipid (top panel). Asterisks indicate endogenous biotin containing protein. Ponceau stain of the biotin blot shown above (bottom panel). [file mmc2.zip › FigureDraft_2014-revised_Page_S2.tiff]
